# Using a modified version of photovoice in a European cross‐national study on homelessness

**DOI:** 10.1002/ajcp.12586

**Published:** 2022-02-09

**Authors:** Marta Gaboardi, Massimo Santinello, Michela Lenzi, Francesca Disperati, José Ornelas, Marybeth Shinn

**Affiliations:** ^1^ Department of Developmental and Social Psychology University of Padova Padua Italy; ^2^ APPsyCI—Applied Psychology Research Center Capabilities and Inclusion ISPA‐Instituto Universitário Lisboa Portugal; ^3^ Department of Human and Organizational Development Peabody College, Vanderbilt University Nashville Tennessee USA

**Keywords:** cross‐national, Europe, homelessness, photovoice, social service providers

## Abstract

This study proposes an innovative use of a modified version of photovoice for cross‐national qualitative research that allows participants to express their ideas, experiences, and emotions about a topic through photographic language. We examine factors affecting social service providers' work on people experiencing homelessness in Europe. We highlight five advantages of using photovoice in cross‐national research: visual language, methodological flexibility, participatory data analysis, the bottom‐up process, and the promotion of social change. Moreover, we identify key stages of the process: writing a detailed protocol for the implementation and fidelity of the projects, using two levels of data analysis, and disseminating the results. This study provides lessons learned for others who may want to use photovoice in cross‐national research.

## Highlights

- Qualitative methods provide a detailed understanding of people's experiences across countries.
- This article offers an example of using photovoice to study homeless services in Europe.
- This study showed the benefit of using photovoice in comparative qualitative research across nations.
- This study provides recommendations for those who may want to use photovoice in cross‐national research.

## INTRODUCTION

In an increasingly multi‐cultural world where interventions transcend national boundaries, international research collaborations involving multiple countries are increasing (Francescato, 2017). The research seeks to make cross‐country comparisons, transfer good practices, and analyze the influence of context on universal and complex phenomena, especially in the social sciences. In Europe, for example, with the European Research Council, funding is growing for research that takes a more global view, for instance, Horizon 2020 projects (Haak et al., 2013). EU‐funded research partnerships require researchers from different nations to collaborate as a condition for securing research funding. Therefore, the research must increasingly consider an interdisciplinary and intercultural vision of the phenomena. Important goals for the partnerships are to understand the extent to which there is a common European culture concerning social issues and for collaboration between countries to promote shared guidelines for intervention and exchange of evidence‐based practices.

Based on our experience in a cross‐national, interdisciplinary European project called HOME_EU: “Homelessness as Unfairness,” we propose an innovative use of photovoice in cross‐national research. The global aim of HOME_EU was to provide an integrated and ecological perspective on homelessness in Europe and to develop guidelines for European countries to address homelessness, supporting the transition from traditional services (TS) to evidence‐based intervention practices such as Housing First (HF; Padgett et al., 2016). Understanding the perspective of social service providers is essential to addressing efforts in this change.

This study aims to show how a modified version of photovoice can be used to understand the perspectives of social service providers engaged in parallel projects across multiple countries and languages. Specifically, we explored the job environments and perceptions of social service providers who work with people experiencing homelessness in eight European countries. We highlight the advantages of using this method in cross‐national studies, describe the steps in the process, and consider some lessons learned for others who wish to use the technique. We believe that some recommendations drawn from our study can contribute to the methodological discussion of cross‐national qualitative research.

### Advantages of a qualitative approach in cross‐national research

Qualitative research aims to capture people's experiences and the meanings they give to their experiences, seeking to better understand social and psychological processes (Willing, 2019). Moreover, qualitative research allows a deep understanding of the challenges, resources, and problems that confront people in the communities in which they live and are involved (Gómez & Kuronen, 2011).

Qualitative research embodies many of the principles of community psychology: the respect for and emphasis on diversity, the use of an ecological framework, and a focus on empowerment, documenting the voices of marginalized or understudied communities, and respect for *emic* (insider) perspectives (Banyard & Miller, 1998; Brodsky et al., 2017). Both community psychology and qualitative research seek to understand individual and community experiences in local settings and cultural contexts. Thus, the attention to the singularity of individual and collective experiences and the possibility of interpreting data in light of the context in which they are collected make a qualitative approach particularly suitable for the analysis of specific contexts, as in cross‐national research. A qualitative approach can reveal specific and local contexts, unique aspects, and cultural nuances (Gómez & Kuronen, 2011; Mangen, 1999); it can also help to understand commonalities across local contexts.

Recently, qualitative methods have been used in cross‐national research to understand and compare different cultural contexts (Bird et al., 2013; Mangen, 1999), sometimes in combination with quantitative methods (Hantrais, 2005; Hines, 1993). Qualitative methods provide the opportunity to gain a detailed understanding of people's experiences, behaviors, and attitudes across countries (Quilgars et al., 2009).

Nevertheless, while quantitative cross‐cultural researchers have developed sophisticated guidelines for translating questionnaires and using confirmatory factor analysis, item analysis, and other tools to establish measurement equivalence across linguistic and cultural contexts (e.g., Beaton et al., 2000; International Test Commission, 2018; Leong et al., 2020; Lynn, 2003), the methodological aspects of qualitative cross‐cultural research lack as much scholarly attention. These shortcomings may due to the challenges of using qualitative methods in cross‐national research.

### Challenges in the cross‐national and multi‐language research

Despite the abovementioned advantages, cross‐national research raises some challenges, especially with qualitative methods. Researchers must interpret information across multiple cultural and socio‐political contexts by collecting information within a framework that is flexible enough to allow for context‐specificity and robust enough to allow cross‐national or cross‐cultural comparisons (Bird et al., 2013; Quilgars et al., 2009). Challenges include sampling, study management, language, and cultural norms (Hines, 1993; Mangen, 1999).

Qualitative cross‐national research samples are typically restricted, drawing the appropriateness of the national units of analysis into question (Mangen, 1999). Even if generalization is not the aim of qualitative research (Fine, 2006), the problems of representativeness of the phenomena under study are important. Often, choosing a sample is based on practical opportunities and the availability of data (Gómez & Kuronen, 2011). Some studies have used purposive sampling procedures (Quilgars et al., 2009) or the “snowballing” technique (Mangen, 1999) to overcome this limitation.

Another challenge is the diversity of cross‐national teams. In cross‐national studies, each team brings different theoretical, cultural, and linguistic backgrounds and different methodological and research experiences (Haak et al., 2013). Since the interpretation of data is a key element in qualitative research, it is necessary to have similar starting points for terminological issues and methodology. In Europe, researchers have taken different approaches to this challenge, such as the development of a common structure in a workshop (Bird et al., 2013) or multiple international workshops and training courses in all countries managed by the project leader before the research team members conduct the methodology (Haak et al., 2013). A collaborative and flexible stance throughout the research project is necessary, with ongoing support provided to each country to adapt and implement the study, including email and phone support (Bird et al., 2013). In Quilgars et al.'s (2009) study, data were analyzed through the production of country reports by each team, followed by a cross‐country comparison with a grounded approach led by two research institutes. Their study benefited from a framework that allowed researchers to grasp the meaning of national policies, thanks to the effective collaboration of team members across eight countries.

Qualitative cross‐national research implies learning across different languages, cultures, and differences in conceptualization. The linguistic problem extends beyond different spoken languages to different meanings and cultural norms (Gómez & Kuronen, 2011). Some researchers benefit from using a common framework and qualitative data analysis software because both provide structures to aid comparability and adaptation at the country level. Using qualitative data analysis software and a framework approach may facilitate analysis (Bird et al., 2013; Haak et al., 2013). This approach may facilitate retaining analysis within the researchers' native languages for as long as possible, combining memo writing to determine meanings for concepts used (Haak et al., 2013). Moreover, expert cultural or ethnic consultants may be involved in evaluating the translation and interpreting the data, thus providing the cultural context for interpreting the responses of specific groups or ethnic minorities (Okazaki & Sue, 1995). Despite these challenges, the reviewed studies highlighted the importance of a collaborative and equitable research process and the need for transparency in reporting the methods used for cross‐national research (Bird et al., 2013).

Recognizing the potential of photographic language to overcome some of the main limitations of cross‐national research, we propose the potential power of a modified version of photovoice for research involving multiple countries and languages.

### Photovoice in cross‐national research

In photovoice, participants take pictures illustrating particular issues, emotions, or experiences, share them in groups, and discuss them to identify themes. When used as part of a participatory action research project, group members translate their themes into practical proposals for social change that they share with the community and policymakers (Pruitt et al., 2018; Wang, 2003; Wang & Burris, 1997).

In community psychology, photovoice has been used because it encourages participants to stand up for the issues they consider important, thus growing a critical consciousness (Carlson et al., 2006) through group discussion to move toward social action (Rania et al., 2015). This method integrates photography and critical discussion to examine topics from the perspective of the participants who are considered experts in the environments in which they live (Wang, 2003). Few studies have used photovoice in cross‐national studies (Fernandes et al., 2018; Malherbe et al., 2017). Nevertheless, photovoice has been used to investigate the role of culture, cultural differences between countries, and the method's adaptability to different settings (Castleden & Garvin, 2008; Teti & van Wyk, 2020).

Photovoice is ideally suited to cross‐national qualitative research for five reasons. First, the photovoice uses visual language. Study in multiple cultures acknowledges the ability to interact with images (Boydell et al., 2012; Moxley & Calligan, 2015). Photographs ease and enrich participants' verbal descriptions of their experiences, leading to a better understanding of people's personal and collective realities (Burles & Thomas, 2014; Foster‐Fishman et al., 2005). Shooting a photo helps one express something that may be hard to put into words. By describing their pictures, participants expressed their personal perspectives, values, ideas, emotions, and experiences (Rania et al., 2015). The photographic language is equal and universal, representing an efficient way to communicate across cultures and social classes. The photovoice was pioneered in studies with marginalized populations (Wang & Burris, 1994; Wang et al., 2000).

Second, the photovoice is flexible. Although it is a well‐defined method, with fundamental steps (Wang & Burris, 1997), it retains the flexibility typical of participatory action research methods. The method can be adapted to suit the needs of participants in the research process, adapting to different community contexts, personal characteristics, and research interests (Catalani & Minkler, 2010). Different studies have highlighted the effectiveness and feasibility of this methodology in different contexts, documenting the adaptability of the methodology to different cultures and countries (Catalani & Minkler, 2010; Hergenrather et al., 2009; Seitz & Strack, 2016).

Third, in the photovoice process, participants share and analyze the photographic data they produce (e.g., Freedman et al., 2014). Frequently, images are metaphors for life experiences and/or emotions. People can represent an idea by using photos of inanimate objects as symbols of their personal representation of a topic (Rania et al., 2015).

Participants' interpretations of the images are particularly important for understanding the meaning of photos and managing and informing researchers' expectations and beliefs, a potential bias in qualitative research (Levitt et al., 2020). In the photovoice process, researchers are facilitators rather than experts. They aim to stimulate critical social consciousness in participants and promote social change in the community involved, but the participants are the real experts of the topic. The themes are often developed with the participants and revised and validated by participants (Hergenrather et al., 2009). This ensures that the analysis is rooted in the language and cultural meanings of the images from participants' perspectives, which, as we explained earlier, is a major challenge in cross‐national qualitative research.

Fourth, photovoice is a bottom‐up process that allows the co‐construction and exploration of new constructs (Plunkett et al., 2013). Thus, themes and knowledge of unexplored contexts can be constructed through images. In this sense, a photovoice can be used as a qualitative method to increase knowledge on a topic (Willig, 2019). Photovoice provides an opportunity to learn and analyze unexplored contexts through the participants' interactive process of developing and constructing meaning from their experiences. Researchers and community members become co‐learners, bridging cultural differences and sharing expertise based on professional knowledge and participants' experiences (Hergenrather et al., 2009). Photovoice can also explore the transferability of theories and themes or other research findings to other contexts, for example, across countries.

Finally, photovoice fosters change in participants (Foster‐Fishman et al., 2005) and the community being investigated (Suprapto et al., 2020). In addition to promoting knowledge, photovoice may be considered an intervention method at the local level to promote social change (Wang, 2003). As a community‐based participatory research method based on feminist theory, constructivism, and documentary photography, photovoice enables participants to engage in personal and community changes (Hergenrather et al., 2009). It promotes social participation and encourages participants to collaborate to define problems, collect information, and use the knowledge to promote social change in their community (Rania et al., 2015; Suprapto et al., 2020). For cross‐national research, these last two advantages allow for both a local level of social change and a broader one based on knowledge creation and comparison of the countries involved.

The remainder of this article describes a European study using a modified version of photovoice across eight countries and languages, where no researcher spoke even half of the languages involved. We depict the challenges in four key stages: writing of a detailed protocol, implementation and fidelity of photovoice projects, data analysis, and results, with examples of how these stages were undertaken in practice. The full participatory action research potential of traditional photovoice has not always been realized in local contexts. However, even where this was not the case, the project allowed understanding and analysis of parallels in the experiences of participants across countries. Findings from all countries contributed to an international dissemination effort. The concluding section considers lessons learned and ways to improve the use of this methodology in future cross‐national research.

## PROCEDURE

### Context of the current research

The current research is part of the cross‐national, interdisciplinary European project HOME_EU: “Homelessness as Unfairness.” It was a three year (2016–2019) multi‐method project funded by the program “Horizon 2020” (Ornelas et al., 2021). The countries involved were France, Ireland, Italy, the Netherlands, Poland, Portugal, Spain, and Sweden. The project aimed to provide a comprehensive understanding of European homelessness by analyzing multiple points of view, such as that of citizens, policymakers, people experiencing homelessness, and social service providers. The European Ethics Committee (Ref. Ares (2017) 535021‐31/01/2017) and the Ethics Committee of each University/Research partner of the HOME_EU Consortium approved this research. Each national research team was responsible for guiding a different aspect of the work and for collecting data with respect to every aspect in their own country.

The social service providers' study was designed to explore factors affecting social service providers' work in homeless services. In a two‐step process, we used both quantitative and qualitative methods (Disperati et al., 2021): the present paper focuses on photovoice and focus groups (Gaboardi et al., 2019) to explore social service providers' experience in homeless services. We also developed a quantitative tool for the organizational analysis of homeless services, as described elsewhere (Gaboardi et al., 2020; Lenzi et al., 2021). In addition to the advantages of the photovoice explained above, this methodology was also consistent with the overall goal of the larger project: to have a European understanding of homeless services from the perspective of social service providers and to promote social change from an ecological perspective.

This study focuses on how a modified cross‐national version of photovoice is a useful qualitative research method to facilitate understanding across multiple countries and languages, as well as lessons learned in the implementation of the process. Specific analyses of the results of the photo projects have been reported elsewhere (Gaboardi et al., 2022).

As community psychologists, at the country level, we used the photovoice methodology proposed by Wang and Burris (1997) in accordance with the principles of participatory action research. Despite this, as we will see in the results and discussion sections, most countries did not take full advantage of the potential of photovoice to facilitate action, as not all countries have implemented social change actions in the organizations involved. We analyzed data at the cross‐national level in the second phase and used photovoice as a method to understand a common European culture of homeless services and to contribute to the elaboration of policy guidelines shared among countries. We utilized both the participants' photographs and narratives and the researchers' interpretation (Tsang, 2020).

### Designing the research protocol

**Figure 1:**
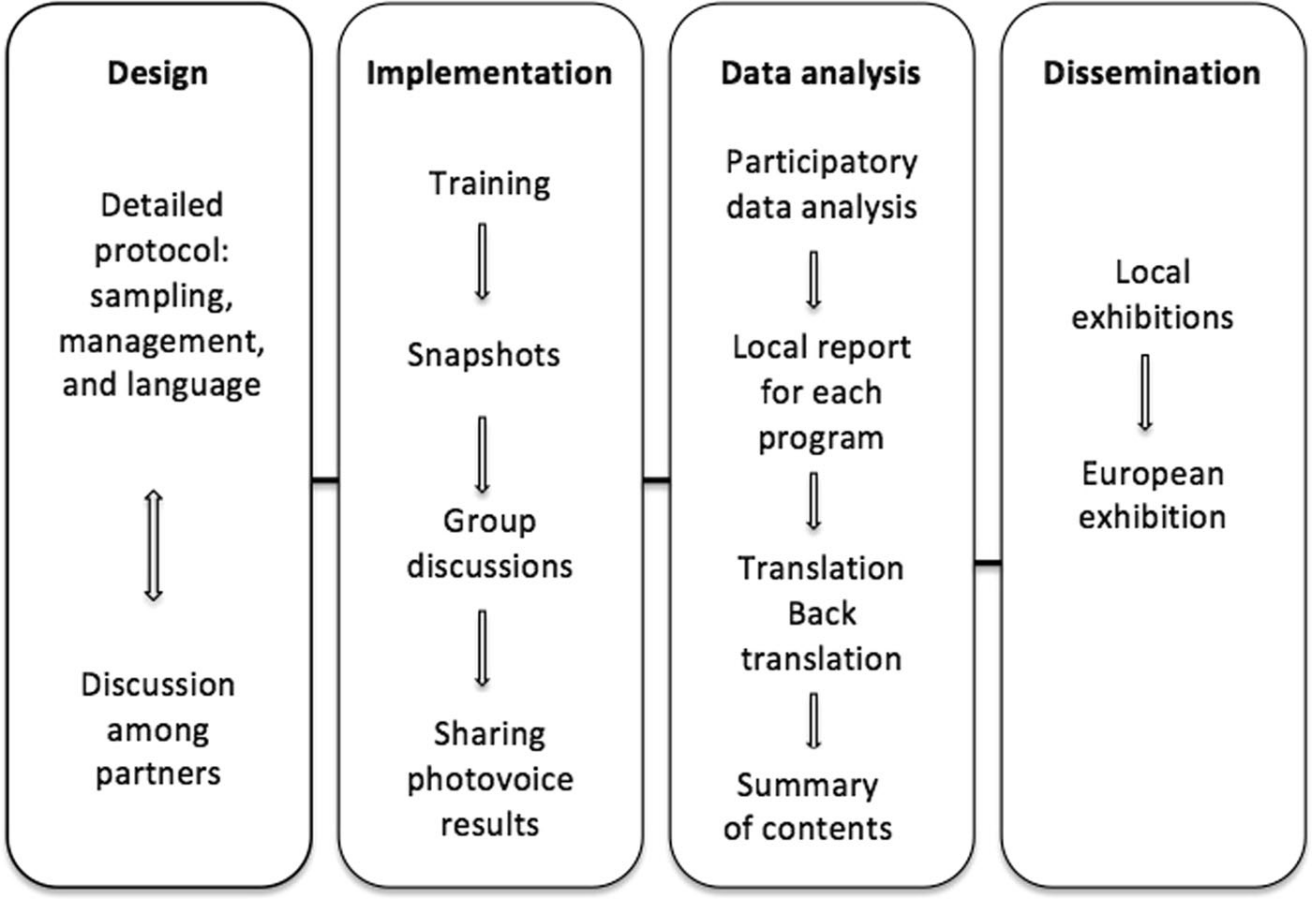
Photovoice process in a cross‐national research

At the beginning of the HOME_EU project, for the social service provider's study the Italian team developed a detailed protocol for implementing the photovoice projects, as summarized in Figure 1. Since the country's partners had no experience with photovoice and had different backgrounds, we provided each country team with practical guides in addition to the protocol to learn about photovoice in detail (Palibroda et al., 2009). The protocol was shared via email with HOME_EU research partners in all countries and discussed during one of the consortium's regular biannual meetings.

Starting with the overall research goal of analyzing factors that influence social service providers' work with people experiencing homelessness, the protocol specified three fundamental aspects of cross‐national qualitative research (Mangen, 1999): a sampling of organizations and participants, project management, and language.

#### Sampling methods

The HOME_EU partners in each country selected organizations that worked with people experiencing homelessness. Although sampling was based on availability, we recommended specific criteria for the selection of organizations and participants. When, due to peculiarities of services in a specific country, it was not possible to respect one or more of these criteria, we asked partners to provide information about the choices made about sampling. The criteria were as follows:

- Type of services: both TS and HF programs were involved in the research (Gaboardi et al., 2019), with at least one program of each type.
- Geographical location: when possible, sampled organizations of each type should be in the same cities (or geographic areas).
- The number of service providers: ideally, at least four staff members from each program should be involved.
- Length of service: only staff members who have worked in the organization for at least 6 months (so that they had enough work experience to report on) and who worked together as a team, meeting regularly should be included.
- Direct service: participants should be engaged in direct service with users, not merely administration.

Before proceeding with the photovoice projects, we asked partners to tell us about any issues and update us on sampling and photovoice meetings. Throughout the project phase, we stayed in touch with HOME_EU Consortium partners via email, and we discussed projects together in face‐to‐face meetings every six months. One photovoice project in an HF service and a photovoice project in TS were conducted in most countries. Exceptions were Italy, where two HF and one TS photovoice projects were conducted, and Poland, where two TS photovoice projects occurred since there were no HF programs in the country yet.

#### Ethical issues

For each phase of the research, we provided templates of consent forms in English, which were translated and adapted by partners based on their country's laws. Informed consent forms were provided for the organizations involved, the research participants, and any individuals who could be photographed by the participants (to give consent to be photographed and to use the photograph in the project).

Next, the protocol detailed the procedure for the photovoice groups, as summarized in Table 1, giving instructions about the necessary steps of typical photovoice projects. All groups were conducted in the local language.

**Table 1:** Summary of the photovoice protocol for cross‐national research

Weeks	Phases	Photovoice activities	Cross‐national considerations
1	Sampling	Recruiting organizations Ethical issues	Recommending specific criteria in the selection of participants; Provide informed consent templates to be translated and adapted to each country's laws.
2	Beginning	Establish the photovoice group Give the consent forms to organizations	Share practical guides on how to conduct photovoice.
3	Photovoice Meetings	(a) Introduction and review of the project with participants; Discussion about power and ethics (b) Picture training (with local photographer); Assign the photographic task (c) Sharing and discussing photos using SHOWeD method; Assign the second photographic task (optional) (d) Sharing and discussing photos; Write the caption and title of the photos (e) Data analysis with participants; Create a draft summary report with participants (f) Discuss a summary report with the participants; Provide a printed copy of the report to each ones	Staying in touch with partners via email or conference call to discuss possible protocol changes or get feedback on how projects are going; The draft country reports should be reviewed within the group in the native language to ensure consistency of cultural meanings in the English translation.
4			
5			
6			
7			
8			
9	Photovoice Dissemination	Organize and promote the exhibition; Involve providers, politicians, mass media and citizens; Promote social change at the local level (e.g. using local results for advocacy).	The translation of country report should follow best practices procedures (Beaton et al., 2000); A cross‐national synthesis should be based on themes chosen by the groups in each country and shared with group facilitators; Develop cross‐national policy guidelines; Dissemination of results (e.g., exhibition, conference, and report) at the cross‐national level.

#### Language and translation

The translation procedure followed the best practices (Beaton et al., 2000). The draft country reports were reviewed with the group in the native language, further refined, and discussed until a consensus was reached. This process maintains participant involvement in the analysis process and ensures the consistency of cultural meanings in English translation. Then, the local research partners who facilitated the photovoice discussion translated the photo captions and the report of themes from the national language into English as the *lingua franca* for the project. Then, professional translators performed back translations, which were checked by the local research partners. Translations were modified as necessary so that the original meaning and cultural nuances were retained.

### Implementation

Overall, 17 photovoice projects involving 81 social service providers in eight European countries were conducted, and 195 photos (HF = 97, TS = 98) were included in the data analysis.

All countries successfully implemented the phases of the photovoice, producing rich data for cross‐national analysis. It was impossible to find organizations willing to participate in more than three meetings in one country. Therefore, both groups had three face‐to‐face meetings, and the sharing of results was done online. The changes were not as disruptive as to be considered in the analysis. We had no other reports of major changes to the protocol concerning photovoice meetings, but in most countries, groups stopped after analyzing their data and preparing a report, rather than using photovoice to move to action. Thus, the lack of social change actions could be because, at the country level, photovoice was considered more as a research method to investigate obstacles and facilitators of social service providers' work and not as a participatory action research methodology. As we will see in the results, in only three countries, the groups present their results to politicians and citizens. In one country, the project facilitated advocacy with respect to a new model of homeless services, and another participant developed operational proposals for change in their own organizations.

Moreover, cross‐national data has enabled the identification of a European culture of homeless services and the development of policy guidelines to assist in transitioning from TS to evidence‐based programs such as HF.

### Data analysis

First, data analysis was conducted within each country and within each photovoice group in collaboration with participants (Powers et al., 2012). Participants generated photos, titles, and captions, and were encouraged to think about themes related to the pictures. Participants decided on the main themes discussed by the group. Facilitators created a draft report incorporating all the themes discussed until saturation, and presented this to the group. During the final meeting, the draft report with all the themes was again reviewed within the group and modified until it was approved by everyone.

For the cross‐national analysis, we added a phase to the photovoice process with an analysis built on the country‐level thematic analysis (Tsang, 2020). We asked all partners to send us photos that best represented each of the themes discussed in each project. Overall, 195 photographs were related to 17 themes identified by participants at the country level. Then, all 17 themes were summarized at the systemic, organizational, and individual levels according to an ecological perspective (Bronfenbrenner, 1977) by three independent Italian researchers (a doctoral student, a research fellow, and a full professor). To ensure consistency of themes in the analysis of aggregate data, we reported all the themes discussed in each country in the aggregate project report with the idea of having a European vision of homeless services. The report was then shared and discussed with all consortium partners, who included the group facilitators, to have a member check that we had interpreted the themes in a way that was consistent with each country's culture.

#### Sample results: similarities and differences between countries

**Figure 2:**
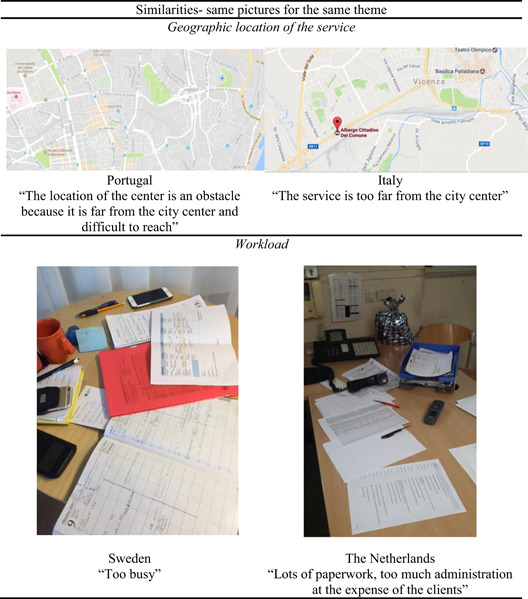
Examples of similarities between countries

The synthesis of results across nations allowed us to envision some similarities immediately. We were surprised to see that some photos were almost identical, despite being shot in different countries, as shown in Figure 2. These included dissatisfaction with the location of the service program (shown with maps) and with the workload, including paperwork (shown with pictures of desks strewn with papers).

**Figure 3:**
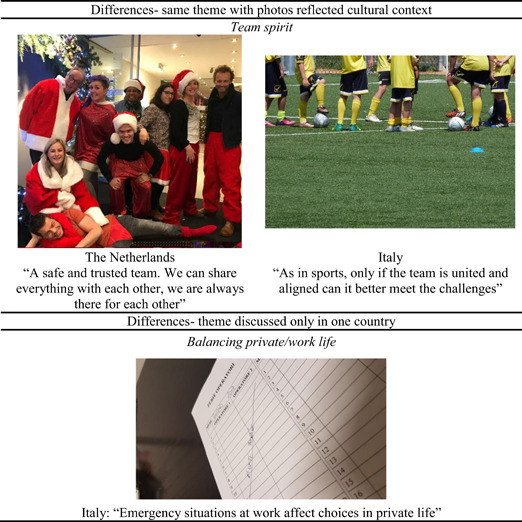
Examples of differences between countries

Sometimes the same theme, such as team spirit among colleagues, was illustrated with country‐specific photos, as shown in Figure 3. In Italy, a participant photographed a soccer team (Italy's most popular sport), while in the Netherlands, colleagues dressed as Santa Claus, a name with a Dutch etymology. Nevertheless, since neither soccer nor Santa Claus is confined to their countries of origin, participants may have used these symbolic photographs to convey a globally relevant message that goes beyond local relevance.

Finally, some topics were discussed in only one country, as shown in Figure 3. For example, the balance between work and private life, into which work emergencies intrude, was mentioned in Italy, but not elsewhere. For a full description of the 17 themes, see Gaboardi et al., 2022.

#### Researchers reflexivity

Some aspects of researchers' backgrounds may have influenced the results. The researchers involved in this project were all from different backgrounds (community psychology, medicine, social sciences), and this may have also influenced the style of conducting the photovoice projects. In addition, only the lead group in the study had experience with the photovoice methodology. Even though all team partners were experienced in homelessness, this was the first time they had used photography methodology. The lack of effort in producing social change may also be due to facilitators' lack of background and experience with participatory action research methodologies in most countries.

## RESULTS

The results of this study can be summarized in two main aspects:

### Social change and local exhibitions

In Italy, Poland, and France, participants and researchers organized exhibitions in strategic places to present the photovoice results to the community and local politicians. These exhibitions have been an opportunity to raise community awareness of homelessness. The exhibitions were visited by citizens, providers in other community services, mass media, and local politicians. As is often the case with a photovoice project, dissemination has led to a change. For example, in an Italian photovoice project, the participants elaborated four proposals for change in their organization: internal communications, the relationship between providers and volunteers, the need for constant updates on the clients' process, and training and psychosocial supervision of the team. Photovoice results and proposals were presented during a meeting with the organization's leaders and shown in an exhibition associated with a conference with citizens and providers from other services. In a follow‐up evaluation one year later, most of the proposals identified through the photovoice had been implemented. Further, in Poland, reflection on photos contributed to the spread of advocacy initiatives that have led to the implementation of HF (Bokszczanin et al., 2021).

### Increasing knowledge about a European vision of homeless services

The topics discussed by participants contributed to a European view of homeless services and the differences between TS and HF (Gaboardi et al., 2022). The themes discussed by the participants contributed to the development of European policy guidelines for homeless services in Europe [D7.4 Policy Guidelines; Ref. Ares (2019) 7374716—29/11/2019]. Moreover, selected photos from all the projects were presented in an exhibition called “Working with homelessness” and hosted at the Municipality Center in June 2018 in Padua, Italy, as part of the 3^rd^ International HF Conference. The exhibition, combined with the conference on the topic, was an opportunity to showcase international comparisons with an international audience. In addition, the results were collected in a book that was distributed during conferences and seminars related to the HOME_EU project (Santinello et al., 2018).

## DISCUSSION

The present study illustrates a novel method for conducting qualitative research across eight nations with eight different languages. To our knowledge, this is the first study to use photovoice in cross‐national research in Europe.

The method takes advantage of the visual language of photography, allows flexibility in implementation. Through a bottom‐up process and participatory data analysis, the method explores participants' ideas, experiences, and emotions. The benefits of using photovoice in cross‐national research have been confirmed in the study. First, visual language helped get social service providers to talk about their work contexts. With photographs, they spoke not only about aspects of the organizations but also about their struggles in working with people experiencing homelessness and challenges with the larger community (e.g., citizens and politics), while also bringing out their emotions (Gaboardi et al., 2022). As Figures 2 and 3 show, the visual language allowed us to observe the similarities and differences between countries. Although the eight countries involved in this study spoke eight different languages, cultural contexts across mostly Western European countries probably vary less than would be the case for countries sampled from different continents where the risks and consequences of speaking up might vary. Moreover, depending on the range of cultures included in a cross‐national study, additional efforts might be necessary to help participants have confidence in the research process.

Second, as shown in other studies (Catalani & Minkler, 2010), each state adapted the photovoice. There were no particular difficulties in implementation, but the benefit of promoting social change at the local level was only partially achieved. Not all local projects have developed proposals for changes in their organizations. Perhaps the goal of social change was too ambitious and expensive (in terms of time and resources) compared to the timeframe of the HOME_EU project. The three year project required several research steps in a short period and with tight deadlines. This may have led to a preference for data collection for cross‐national studies rather than focusing on the process of social change at the local level.

Third, data analysis with participants allowed for consistent interpretation of the images, overcoming interpretation bias by researchers with different cultures. Participants elaborated the themes so that the cultural significance of the photographs was made explicit and maintained in the cross‐national comparison. Fourth, this study led to a greater understanding of homeless services in Europe and the development of both factors that foster and hinder work with people experiencing homelessness (Lenzi et al., 2021; Gaboardi et al., 2022). The cross‐cultural project allows a comparison of participants' experiences in different countries. Although generalization is not the aim of qualitative research, and country‐to‐country differences in themes may reflect local sampling or other differences in implementation, the recurrence of some themes across countries suggests commonalities across countries in the experiences of social service providers in the homelessness sector. This is especially important for elaborating European guidelines to combat homelessness and, therefore, to promote European funds to support innovative services that help professionals in their work with people experiencing homelessness. For example, many European countries are moving to adopt HF approaches to homelessness based on evidence of the superiority of HF over TS generated in the United States (Padgett et al., 2016), Canada (Aubry et al., 2015), France (Tinland et al., 2013), and by the HOME_EU project (Ornelas et al., 2021). Understanding the perspectives of social service providers is essential for successful change. The cross‐national study uncovered some common and more local challenges that must be addressed as part of this cross‐national change effort. As Fine (2006) noted, to enhance the theoretical generalizability of a study, researchers from different contexts should create opportunities to discuss the similarities and differences of the social issues investigated together. However, these conversations should not be limited to presenting research findings in different contexts but should explore which aspects are most prominent in each context to find the best solutions to support the work of policy, activism, and practice.

Regarding the fifth benefit of promoting social change, only three countries disseminated the results with the community, and only one (Italy) documented long‐term changes in an organization involved. More training to partners on the guiding principles of the methodology and the importance of participatory action research could have helped them understand the potential of photovoice. The project also contributed to change at a cross‐national level by contributing to the development of European guidelines for implementing services to tackle homelessness [D7.4 Policy Guidelines; Ref. Ares (2019) 7374716—29/11/2019].

Social change is the most fundamental aspect of photovoice, but it is also the most difficult to achieve because it requires time, resources, and a reflective approach from researchers (Malherbe et al., 2017). The cross‐national photovoice project can be thought of as a multi‐level intervention that can facilitate change at both the local and international levels, even if the potential for change was not fully realized.

## LIMITATIONS, LESSON LEARNED, AND RECOMMENDATIONS FOR FUTURE RESEARCH

During the study process, we encountered some challenges and learned some useful lessons for those who wanted to use this methodology in the future. These lessons cover four main aspects: (a) support in the process, (b) promotion of social change, (c) analysis of photographs, and (d) the comparison between nations.

First, regarding the support in the process, this research was made possible by the continual interactions among the project's consortium members and was based on a common detailed protocol about planning (aims, recruitment, setting, role of the facilitators, ethics), process (detailed explanation of each step of the photovoice method), and data analysis. This ensured that the research was methodologically solid, balancing the need for the research to be structured enough to allow cross‐national comparisons while ensuring that it was grounded in local contexts. Nevertheless, it was sometimes difficult to know in detail how photovoice projects were progressing. In addition, the country reports of themes varied considerably in length across the photovoice groups. It is possible that groups with short reports also discussed some of the themes apparent in the lengthier reports, but did not deem them sufficiently central to mention. It is also possible that facilitators who drafted the reports and translated them into English had different understandings of the amount of detail it would be valuable to include, or, because of differential levels of comfort in English, chose shorter or longer presentations.

Moreover, regarding the second lesson learned, we note that not all countries fully realized the social change goal of photovoice. Future studies using this methodology would benefit from a better balance between research and action by using research findings to inform organizational and community changes based on scientific knowledge. Greater support in learning about the guiding principles of the methodology and how to support social change might have helped more countries realize this goal (e.g., with examples from previous projects).

In the future, we suggest starting with a workshop—at least a one day workshop (Bird et al., 2013)—with all partners to explain the methodology and its principles in detail. We will include examples of photos, discussions, and previous projects, highlighting the process of social change for participants and the community. Moreover, providing facilitators with more guidance on how much to include in reports of themes through a predefined report template might lead to more uniformity. It would also be useful to conduct periodic online meetings to support partners step by step, especially in disseminating results at the local level and elaborating operational proposals by the group of participants.

Regarding the analysis of the photographs, that is, the third lesson learned, it is important to note that the analyst's culture might affect the interpretation of the images. Both the discussion of themes between the facilitator and participants and the discussion among partners were critical to foster an understanding of the results. The captions that participants provided for their photos were also important. Without text, we would have interpreted the figures differently because they were tied to the cultural context. With the translation of the texts, we hope that we have interpreted photos in ways that are faithful to participants' ideas and emotions.

Finally, the fourth lesson concerned the analysis of cross‐national comparison data. The cross‐national comparison (i.e., overall results) was not shared with all photovoice project participants. Only a few participants saw the European exhibition in Padua. Member‐checking is an important way to assure the trustworthiness of qualitative research (e.g., Lincoln & Guba, 1985). Member‐checking at the cross‐national level was confined to the facilitators of each group. In the future, those who want to use cross‐national photovoice could share the overall results with all participants to receive additional feedback. Doing so in our case would have required translation back into eight local languages.

We have encountered some challenges commonly found in multi‐country research with the researchers themselves coming from different contexts and backgrounds. Based on our experience, we have developed four main recommendations. First, it is important to share the principles of photovoice, not just the methodology, discussing with the researchers the process of photovoice, and how to help the group of participants promote social change proposals in their own context and the larger community so as not to let the cross‐national synthesis prevail at the expense of social change at the local level. Second, we suggest providing local researchers with a country report template containing the results of the project and another one describing reflections of the researchers who conducted the project and explaining participants' proposed social change proposals.

Third, in the data analysis, conducting frequent interpretation sessions with all the partners is necessary to discuss key concepts in‐depth, provide a way to understand national differences, and deepen the meaning emerging from data. Finally, engaging all photovoice participants to confirm cross‐national results would be ideal, in accordance with the principles of participatory action research, for example, through cross‐national reports or conferences with group discussions.

## CONCLUSION

Starting from our research experience with the European project HOME_EU, which aimed to study homelessness in Europe, the main goal of this study was to show how a modified version of photovoice could be used in projects with multiple countries and languages. In this article, we started by reflecting on the increasing diffusion of cross‐national research, especially in the European context, which aims to understand the extent to which there is a common European culture concerning social issues (such as homelessness), and to promote shared guidelines for intervention between countries.

As we pointed out in the introduction, cross‐national research poses some important challenges that concern sampling, study management, language, and cultural norms. As a participatory action research methodology that uses photographic language, photovoice can overcome these challenges through the use of visual language, flexibility, data analysis with participants, bottom‐up processes, and promotion of social change.

Through our experience in social service providers of homeless services in eight European countries, we have reflected on the feasibility of using photovoice to increase knowledge on a European phenomenon, highlighting some strengths and providing recommendations for those who would like to use this method at a cross‐national level. Our recommendations start with the reflections made during the implementation of the European project and concern. The reflections include the following:

- The importance of sharing the guiding principles of photovoice, not just the methodology to promote social change;
- The need to provide local researchers with both a country report template that contains the results of the project and one to describe reflections of the researchers who conducted the project;
- The need to conduct frequent interpretation sessions with all the partners to discuss key concepts in‐depth;
- The importance of engaging all photovoice participants for confirmation of cross‐national results.

Overall, the implementation of photovoice projects is not only about the adaptability of a method but also the reflective capacity of researchers on the importance of using this methodology to promote social change.

In conclusion, implementing a photovoice in a cross‐national context involves challenges. Nevertheless, the study demonstrated the benefits and feasibility of using photographs in comparative qualitative research across nations, languages, and cultures, not only to facilitate local change but also to understand a common European culture of a phenomenon and to contribute to the elaboration of policy guidelines shared among countries.

## FUNDING INFORMATION

This research was funded by the European Commission through a grant, as part of H2020 research project HOME_EU: Reversing Homelessness in Europe H2020‐SC6‐REVINEQUAL‐2016/GA726997. The grant agreement between the European Commission and each research unit involved allows complete liberty and autonomy of researchers in the design of the study; the European Commission will take no part in data collection procedures, analyses, interpretation of the data, or decision to submit results.

## CONFLICT OF INTERESTS

The authors declare no conflict of interests.

## Data Availability

The data that support the findings of this study are available from the corresponding author upon reasonable request.

## References

[ajcp12586-bib-0001] Aubry, T. , Nelson, G. , & Tsemberis, S. (2015). Housing first for people with severe mental illness who are homeless: A review of the research and findings from the at home—Chez soi demonstration project. The Canadian Journal of Psychiatry, 60(11), 467–474. 10.1177/070674371506001102 26720504 PMC4679127

[ajcp12586-bib-0002] Banyard, V. L. , & Miller, K. E. (1998). The powerful potential of qualitative research for community psychology. American Journal of Community Psychology, 26(4), 485–505.

[ajcp12586-bib-0003] Beaton, D. E. , Bombardier, C. , Guillemin, F. , & Ferraz, M. B. (2000). Guidelines for the process of cross‐cultural adaptation of self‐report measures. Spine, 25(24), 3186–3191. 11124735 10.1097/00007632-200012150-00014

[ajcp12586-bib-0004] Bird, P. , Campbell‐Hall, V. , Kakuma, R. , & MHaPP Research Programme Consortium . (2013). Cross‐national qualitative research: The development and application of an analytic framework in the mental health and poverty project. International Journal of Social Research Methodology, 16(4), 337–349. 10.1080/13645579.2012.709802

[ajcp12586-bib-0005] Bokszczanin, A. , & Rogowska, A. M. (2021). From evidence to practice: Implementation of the Housing First program in Poland. In J. Ornelas , M. J. Vargas‐Moniz , & HOME_EU consortium study Group (Eds.), *Homelessness as Unfairness*. H2020_HOME_EU: Reversing Homelessness in Europe GA/726997 (pp. 99–106). ISPA—Instituto Universitário. ISBN: 978‐989‐8384‐62‐1.

[ajcp12586-bib-0006] Boydell, K. , Gladstone, B. M. , Volpe, T. , Allemang, B. , & Stasiulis, E. (2012). January the production and dissemination of knowledge: A scoping review of arts‐based health research, In Forum Qualitative Sozialforschung/Forum: Qualitative Social Research 13, No. 1.

[ajcp12586-bib-0007] Brodsky, A. E. , Mannarini, T. , Buckingham, S. L. , & Scheibler, J. E. (2017). Kindred spirits in scientific revolution: Qualitative methods in community psychology. In M. A. Bond , I. Serrano‐García , C. B. Keys , & M. Shinn (Eds.), APA handbook of community psychology: Methods for community research and action for diverse groups and issues (pp. 75–90). American Psychological Association. 10.1037/14954-000

[ajcp12586-bib-0008] Bronfenbrenner, U. (1977). Toward an experimental ecology of human development. American Psychologist, 32(7), 513–531. 10.1037/0003-066X.32.7.513

[ajcp12586-bib-0009] Burles, M. , & Thomas, R. (2014). “I Just Don't Think There's any other Image that Tells the Story like [This] Picture Does”: Researcher and Participant Reflections on the Use of Participant‐Employed Photography in Social Research. International Journal of Qualitative Methods, 13(1), 185–205. 10.1177/160940691401300107

[ajcp12586-bib-0010] Carlson, E. D. , Engebretson, J. , & Chamberlain, R. M. (2006). Photovoice as a social process of critical consciousness. Qualitative Health Research, 16(6), 836–852. 10.1177/1049732306287525 16760539

[ajcp12586-bib-0011] Castleden, H. , & Garvin, T. (2008). Modifying Photovoice for community‐based participatory Indigenous research. Social Science & Medicine, 66(6), 1393–1405. 10.1016/j.socscimed.2007.11.030 18191883

[ajcp12586-bib-0012] Catalani, C. , & Minkler, M. (2010). Photovoice: A review of the literature in health and public health. Health Education & Behavior, 37(3), 424–451. 10.1177/1090198109342084 19797541

[ajcp12586-bib-0013] Disperati, F. , Gaboardi, M. , Lenzi, M. , Vieno, A. , & Santinello, M. (2021). New Strategies to Study Organizations Working with People Experiencing Homelessness: The Service Providers' Study. In J. Ornelas , M. J. Vargas‐Moniz, & HOME_EU consortium study Group (Eds.), *Homelessness as Unfairness*. H2020_HOME_EU: Reversing Homelessness in Europe GA/726997 (pp. 57–66). ISPA—Instituto Universitário. ISBN: 978‐989‐8384‐62‐1.

[ajcp12586-bib-0060] Fernandes, H. L. , Cantrill, S. , Shrestha, R. L. , Raj, R. B. , Allchin, B. , Kamal, R. , Butcher, N. , & Grills, N. (2018). Lived experience of psychosocial disability and social inclusion: A participatory Photovoice study in rural India and Nepal. Disability, CBR & Inclusive Development, 29(2), 5–23. 10.5463/DCID.v29i2.746

[ajcp12586-bib-0014] Fine, M. (2006). Bearing witness: Methods for researching oppression and resistance—A textbook for critical research. Social Justice Research, 19(1), 83–108. 10.1007/s11211-006-0001-0

[ajcp12586-bib-0015] Foster‐Fishman, P. , Nowell, B. , Deacon, Z. , Nievar, M. A. , & McCann, P. (2005). Using methods that matter: The impact of reflection, dialogue, and voice. American Journal of Community Psychology, 36(3–4), 275–291. 10.1007/s10464-005-8626-y 16389500

[ajcp12586-bib-0016] Francescato, D. (2017). A critical look at globalization processes and at the internationalization of community psychology. In M. A. Bond , I. Serrano‐García , C. B. Keys , & M. Shinn (Eds.), APA handbook of community psychology: Theoretical foundations, core concepts, and emerging challenges (pp. 485–501). American Psychological Association. 10.1037/14953-025

[ajcp12586-bib-0017] Freedman, D. A. , Pitner, R. O. , Powers, M. C. , & Anderson, T. P. (2014). Using photovoice to develop a grounded theory of socio‐environmental attributes influencing the health of community environments. British Journal of Social Work, 44(5), 1301–1321. 10.1093/bjsw/bcs173

[ajcp12586-bib-0018] Gaboardi, M. , Disperati, F. , Lenzi, M. , Vieno, A. , & Santinello, M. (2020). Working With People Experiencing Homelessness in Europe: A mixed‐method approach to analyse homeless services. European Journal of Homelessness, 14(4), 87–101.

[ajcp12586-bib-0019] Gaboardi, M. , Lenzi, M. , Disperati, F. , Santinello, M. , Vieno, A. , Tinland, A. , Vargas‐Moniz, M. J. , Spinnewijn, F. , O'Shaughnessy, B. R. , Wolf, J. R. , Bokszczanin, A. , Bernad, R. , Beijer, U. , Ornelas, J. , Shinn, M. , & Consortium Study Group HOME_EU . (2019). Goals and principles of providers working with people experiencing homelessness: A comparison between housing first and traditional staircase services in eight European countries. International Journal of Environmental Research and Public Health, 16(9), 1590. 10.3390/ijerph16091590 PMC6539657 31067661

[ajcp12586-bib-0061] Gaboardi, M. , Santinello, M. , Disperati, F. , Lenzi, M. , Vieno, A. , Loubière, S. , Vargas‐Moniz, M. J. , Spinnewijn, F. , Greenwood, R. M. , Wolf, J. R. , Bokszczanin, A. , Bernad, R. , Blid, M. , Ornelas, J. , Shinn, M. , & HOME‐EU consortium study group . (2022). Working with people experiencing homelessness in Europe. Human Service Organizations: Management, Leadership, & Governance , Under review.

[ajcp12586-bib-0020] Gómez, M. V. , & Kuronen, M. (2011). Comparing local strategies and practices: Recollections from two qualitative cross‐national research projects. Qualitative Research, 11(6), 683–697. 10.1177/1468794111413366

[ajcp12586-bib-0021] Haak, M. , Himmelsbach, I. , Granbom, M. , & Löfqvist, C. (2013). Cross‐national and multi‐language qualitative research: Challenges and recommendations. British Journal of Occupational Therapy, 76(7), 333–336. 10.4276/030802213X13729279115059

[ajcp12586-bib-0022] Hantrais, L. (1999). Contextualization in cross‐national comparative research. International Journal of Social Research Methodology, 2(2), 93–108. 10.1080/136455799295078

[ajcp12586-bib-0023] Hantrais, L. (2005). Combining methods: A key to understanding complexity in European societies? European societies, 7(3), 399–421. 10.1080/14616690500194035

[ajcp12586-bib-0024] Hergenrather, K. C. , Rhodes, S. D. , Cowan, C. A. , Bardhoshi, G. , & Pula, S. (2009). Photovoice as community‐based participatory research: A qualitative review. American Journal of Health Behavior, 33(6), 686–698. 10.5993/AJHB.33.6.6 19320617

[ajcp12586-bib-0025] Hines, A. M. (1993). Linking qualitative and quantitative methods in cross‐cultural survey research: Techniques from cognitive science. American Journal of Community Psychology, 21(6), 729–746. 10.1007/BF00942245

[ajcp12586-bib-0026] International Test Commission . (2018). ITC Guidelines for Translating and Adapting Tests (2nd ed.). International Journal of Testing, 18(2), 101–134. 10.1080/15305058.2017.1398166

[ajcp12586-bib-0027] Lenzi, M. , Santinello, M. , Gaboardi, M. , Disperati, F. , Vieno, A. , Calcagnì, A. , Rogowska, A. M. , Wolf, J. R. , Loubière, S. , Beijer, U. , Bernad, R. , Vargas‐Moniz, M. J. , Ornelas, J. , Spinnewijn, F. , Shinn, M. , & HOME_EU Consortium Study Group . (2021). Factors associated with providers' work engagement and burnout in homeless services: A cross‐national study. American Journal of Community Psychology, 67(1–2), 220–236. 10.1002/ajcp.12470 33137234

[ajcp12586-bib-0028] Leong, F. T. L. , Kalibatseva, Z. , & Somaraju, A. (2020). Evaluating Measurement Equivalence in Cross‐Cultural StressResearch. In T. Ringeisen , P. Genkova, & F. Leong , (Eds.), Handbuch Stress und Kultur. Springer. 10.1007/978-3-658-27825-0_13-1

[ajcp12586-bib-0029] Levitt, H. M. , Surace, F. I. , Wu, M. B. , Chapin, B. , Hargrove, J. G. , Herbitter, C. , Lu, E. C. , Maroney, M. R. , & Hochman, A. L. (2020). The meaning of scientific objectivity and subjectivity: From the perspective of methodologists. Psychological Methods. Advance online publication, 1–17. 10.1037/met0000363 33048563

[ajcp12586-bib-0030] Lincoln, Y. S. , & Guba, E. G. (1985). Naturalistic Inquiry. Sage.

[ajcp12586-bib-0031] Lynn, P. (2003). Developing quality standards for cross‐national survey research: Five approaches. International Journal of Social Research Methodology, 6(4), 323–336. 10.1080/13645570210132848

[ajcp12586-bib-0032] Malherbe, N. , Suffla, S. , Seedat, M. , & Bawa, U. (2017). Photovoice as liberatory enactment: The case of youth as epistemic agents, Emancipatory and participatory methodologies in peace, critical, and community psychology (pp. 165–178). Springer International Publishing.

[ajcp12586-bib-0033] Mangen, S. (1999). Qualitative research methods in cross‐national settings. International Journal of Social Research Methodology, 2(2), 109–124. 10.1080/136455799295087

[ajcp12586-bib-0035] Moxley, D. P. , & Calligan, H. F. (2015). Positioning the arts for intervention design research in the human services. Evaluation and Program Planning, 53, 34–43. 10.1016/j.evalprogplan.2015.07.013 26262890

[ajcp12586-bib-0036] Okazaki, S. , & Sue, S. (1995). Methodological issues in assessment research with ethnic minorities. Psychological Assessment, 7(3), 367–375.

[ajcp12586-bib-0037] Ornelas, J. , & Vargas‐Moniz, M. J. (Eds.). (2021). Homelessness as Unfairness. ISPA—Instituto Universitário HOME_EU consortium study Group. Available at: http://loja.ispa.pt/produto/homelessness-unfairness

[ajcp12586-bib-0038] Padgett, D. , Henwood, B. F. , & Tsemberis, S. J. (2016). Housing First: Ending homelessness, transforming systems, and changing lives. Oxford University Press. 10.1176/appi.ps.671101 27903173

[ajcp12586-bib-0039] Palibroda, B. , Krieg, B. , Murdock, L. , & Havelock, J. (2009). A practical guide to photovoice: Sharing pictures, telling stories and changing communities. Prairie Women's Health Centre of Excellence (PWHCE).

[ajcp12586-bib-0040] Plunkett, R. , Leipert, B. D. , & Ray, S. L. (2013). Unspoken phenomena: Using the photovoice method to enrich phenomenological inquiry. Nursing Inquiry, 20(2), 156–164. 10.1111/j.1440-1800.2012.00594.x 22381071

[ajcp12586-bib-0041] Powers, M. , Freedman, D. , & Pitner, R. (2012). From snapshot to civic action: A photovoice facilitator's manual. Community‐Engaged Scholarship for Health (CES4Health). ​ 1–89. Available at https://libres.uncg.edu/ir/uncg/f/M%5FPowers%5FFrom%5F2012.pdf

[ajcp12586-bib-0042] Pruitt, A. S. , Barile, J. P. , Ogawa, T. Y. , Peralta, N. , Bugg, R. , Lau, J. , Lamberton, T. , Hall, C. , & Mori, V. (2018). Housing first and photovoice: Transforming lives, communities, and systems. American Journal of Community Psychology, 61(1–2), 104–117. 10.1002/ajcp.12226 29323410 PMC5891509

[ajcp12586-bib-0043] Quilgars, D. , Elsinga, M. , Jones, A. , Toussaint, J. , Ruonavaara, H. , & Naumanen, P. (2009). Inside qualitative, cross‐national research: Making methods transparent in a EU housing study. International Journal of Social Research Methodology, 12(1), 19–31. 10.1080/13645570701804292

[ajcp12586-bib-0044] Rania, N. , Migliorini, L. , Rebora, S. , & Cardinali, P. (2015). Photovoice and interpretation of pictures in a group discussion: A community psychology approach. Qualitative Research in Psychology, 12(4), 382–396. 10.1080/14780887.2015.1019597

[ajcp12586-bib-0045] Santinello, M. , Gaboardi, M. , Disperati, F. , Lenzi, M. , & Vieno, A. (2018). Working with Homelessness: An European multi‐site photovoice project. CLEUP.

[ajcp12586-bib-0046] Seitz, C. M. , & Strack, R. W. (2016). Conducting public health photovoice projects with those who are homeless: A review of the literature. Journal of Social Distress and the Homeless, 25(1), 33–40. 10.1080/10530789.2015.1135565

[ajcp12586-bib-0047] Suprapto, N. , Sunarti, T. , Suliyanah, D. W. , Hidayaatullaah, H. N. , Adam, A. S. , & Mubarok, H. (2020). A systematic review of photovoice as participatory action research strategies. International Journal of Evaluation & Research and Education, 9(3), 675–683. 10.11591/ijere.v9i3.20581

[ajcp12586-bib-0048] Teti, M. , & van Wyk, B. (2020). Qualitative methods without borders: Adapting photovoice: From a US to South African setting. International Journal of Qualitative Methods, 19, 1–3. 10.1177/1609406920927253

[ajcp12586-bib-0049] Tinland, A. , Fortanier, C. , Girard, V. , Laval, C. , Videau, B. , Rhenter, P. , Greacen, T. , Falissard, B. , Apostolidis, T. , Lançon, C. , Boyer, L. , & Auquier, P. (2013). Evaluation of the Housing First program in patients with severe mental disorders in France: Study protocol for a randomized controlled trial. Trials, 14(1), 1–10. 10.1186/1745-6215-14-309 24063556 PMC3850649

[ajcp12586-bib-0050] Tsang, K. K. (2020). Photovoice data analysis: Critical approach, phenomenological approach, and beyond. Beijing International Review of Education, 2(1), 136–152.

[ajcp12586-bib-0051] Wang, C. (2003). Using photovoice as a participatory assessment and issue selection tool. Community Based participatory research for health, 1, 179–196.

[ajcp12586-bib-0052] Wang, C. , & Burris, M. A. (1994). Empowerment through photo novella: Portraits of participation. Health Education Quarterly, 21(2), 171–186. 10.1177/109019819402100204 8021146

[ajcp12586-bib-0053] Wang, C. , & Burris, M. A. (1997). Photovoice: Concept, methodology, and use for participatory needs assessment. Health Education & Behavior, 24(3), 369–387. 10.1177/109019819702400309 9158980

[ajcp12586-bib-0055] Wang, C. C. , Cash, J. L. , & Powers, L. S. (2000). Who knows the streets as well as the homeless? Promoting personal and community action through photovoice. Health promotion practice, 1(1), 81–89. 10.1177/152483990000100113

[ajcp12586-bib-0057] Willig, C. (2019). What can qualitative psychology contribute to psychological knowledge? Psychological Methods, 24(6), 796–804. 10.1037/met0000218 31008623

